# Looking for the right intention: can neuroscience benefit from the law?

**DOI:** 10.3389/fnhum.2015.00432

**Published:** 2015-08-03

**Authors:** Davide Rigoni, Luca Sammicheli, Giuseppe Sartori

**Affiliations:** ^1^Department of Experimental Psychology, Ghent UniversityGhent, Belgium; ^2^Department of General Psychology, University of PadovaPadova, Italy

**Keywords:** neuroscience, law, intention, free will, control

## Introduction

It is argued that neuroscience will eventually revolutionize the law (Wegner, 2002; Greene and Cohen, 2004; O'Hara, 2004; Churchland, 2011). Virtually all legal systems are grounded on the assumption that human beings (can) act on the basis of their own free will; voluntary actions are driven by people's intentions, and individuals are therefore responsible (and punishable) for their own actions. Neuroscience seems to challenge this assumption at two different levels: at the theoretical or anthropological level by proposing a new model of human being, and at the empirical level by attempting to provide scientific evidence that free will is nothing more than an illusion (Sellars, 1963; Frith, 2007; Churchland, 2011). In the following, we will argue (i) that the anthropological challenge to free will is not specific to neuroscience and (ii) that by focusing on an oversimplified operationalization of free will, current empirical research has only limited impact upon the law. In order to be applicable within the legal system, cognitive neuroscience should rather consider how free will—or the different *modes* of free will—is conceptualized in the legal system, and incorporate these concepts into more appropriate experimental designs.

## The anthropological challenge: neuroscience and the new human being

By explaining voluntary behavior in terms of biological and chemical processes occurring in the brain, neuroscience seems to leave little to no room for free will. This is relevant for the law because if there is no free will, does it make sense to punish people for their crimes? This argument is fuelled by the philosophical position of determinism, which states that every state is entirely determined by preceding physical states that are beyond human control. In a nutshell, the logic goes as follows: since behavior, including our voluntary choices, is generated by a biological machine—i.e., the brain—it necessarily follows the deterministic laws of the physical world. Human behavior is therefore determined in advance by previous states of the world that escape our conscious control. Free will is nothing more than an illusion and individuals should not be held responsible for their actions, not even for the crimes they commit.

Neuroscience is therefore supposed to change the law by offering a different model of human being: according to the law, the human *mind* is generated by the brain but *does not correspond to the brain* and preserves some degree of autonomy; conversely, neuroscience's ultimate goal is to explain the totality of behavior in terms of neural processes (Lavazza and Sammicheli, 2012). From this perspective, although we have the illusion of being in control, all our actions—including crimes—are in fact committed by neural processes that we cannot control (e.g., Wegner, 2002). One should note that this anti-free will position seems very convincing. Indeed, a *brain-centered* vision of the human being has been found to affect the folk psychology of free will: when crimes are described as the result of neural processes occurring in the brain, the attribution of free will and moral responsibility to an hypothetical criminal is reduced (Nahmias et al., 2005, 2007), and people also tend to have less punishing attitudes (Shariff et al., 2014). However, the idea that the causal forces underlying behavior escape human control is not restricted to neuroscience, but derives from the *reduction* of neuroscience in the domain of hard sciences. For instance, behavioral genetics assume that casual DNA variations determine physical characteristics as well as behavioral tendencies (Asbury and Plomin, 2013), including crime-related tendencies such as violence and impulsivity (e.g., Meyer-Lindenberg et al., 2006). In a similar vein, at the end of the nineteenth century criminal anthropologist Cesare Lombroso argued that “*criminals do not commit crimes out of their own free will, but they are urged to commit crimes because of their innate and primitive organic nature*” (Lombroso, 1876, p. 29)^1^. Therefore, the deterministic argument that behavior is governed by factors that are not under human control has been proposed in the past, and it is not specific to neuroscience. In other words, this has nothing to do with the empirical neuroscientific endeavor, but it requires the philosophical endorsement of a specific model of human being.

## The empirical challenge: scientific evidence against free will?

Neuroscience is supposed to influence the law, not just by proposing a different anthropological model of a human being, but also by investigating, at the *empirical* level, the neural bases of human volition. The first attempt to tackle scientifically the problem of free will was conducted in the early ‘80s by Benjamin Libet and his colleagues. In a series of experiments, participants were instructed to perform self-paced voluntary flexions of their index finger while their brain activity was measured with the electroencephalogram (EEG; Libet et al., 1983). While participants decided to press the key they watched a clock hand rotating and were instructed to indicate the moment in time when they formed the intention to move the finger. By comparing the reported moment of the conscious intention “*to move the finger now*” with the EEG signal from motor areas responsible for preparing and executing the movement, it was observed that motor areas in the brain were activated around 1 s before participants reported the intention to move: the brain “knew” that the participant was going to move the finger well before the participant knew himself. According to the authors, this result provided the first empirical evidence that voluntary movements are initiated unconsciously, thereby questioning the traditional concept of free will. Despite some skepticism, the finding that our conscious intentions are preceded by unconscious brain activity in the motor areas of the brain has been replicated (Haggard and Eimer, 1999; Rigoni et al., 2013). The intention “*I want to move my finger now*” would not be the cause of the action, but rather a perception of the current motor state that has already been determined in the brain (Hallett, 2007). One limitation of this type of study is that it only focuses on the *when* component of the action; however, free will also concerns choices, that is, *what* is the specific action we want to perform (Brass and Haggard, 2008). In a few recent studies combining a free-choice paradigm with functional Magnetic Resonance Imaging (fMRI), participants were asked to decide between a left and a right button press that they should execute at a freely chosen time (e.g., Soon et al., 2008). They were then asked to report the time at which the decision was actually made. Results showed that the outcome of the decision was encoded in the brain activity of the prefrontal and parietal cortex up to 10 s before it entered awareness. So the question arises: if we can predict 10 s before someone indicates he is going to press a key which key that will be, then how can we be considered responsible for our own actions?^2^

## Toward a new neuroscience of human volition

While these data are often interpreted as the empirical proof of the view that all the effective mechanisms that cause our actions are unconscious (Wegner, 2002; Hallett, 2007), the key question is whether such empirical evidence can be used by the law to improve the determination of personal responsibility during a trial. In order to be relevant to the law, it is crucial that the type of intention that is investigated in cognitive neuroscience is analogous to the type of intention that is relevant to the law. But is the intention “*to move the finger now*” really paradigmatic of the type of intention that is usually assessed by the Court? How would neuroscience help to establish the culpability related to a murder committed by a serial killer vs. the legitimacy of a “murder” committed by an executioner of a person who is sentenced to death?

In our view, current neuroscientific research on the neural bases of human volition has a limited impact on the law because it focuses on simplified concepts of intention and intentionality. Throughout the centuries, legal systems have developed very sophisticated conceptual categories that are used in Court to determine whether a certain behavior is criminally liable or not. While an exhaustive description of the different categories of intentions and intentionality in the different legal systems obviously is beyond the scope of the present article, here we want to stress that, to be relevant to the law, neuroscience should first endorse more sophisticated and nuanced definitions of intention. In virtually all legal systems, there are at least three categories, or modes, of intentions. The first mode is condensed in the Latin concept of the *suitas*, which refers to the extent to which a certain behavior *belongs psychologically* to the actor in order to be punished. In psychological terms, this means that to be punishable, a behavior has to imply a certain degree of mental presence of the actor. Conversely, an automatism (e.g., a movement triggered by a Transcranial Magnetic Stimulation (TMS) pulse over the primary motor cortex, or a crime committed in a state of somnambulism) does not have any criminal relevance because the behavior does not belong to the actor. In other words, there is no crime if there is no *suitas*^3^. This mode of intentionality therefore qualifies the difference between actions as purely mechanical phenomena—e.g., raising a hand—and actions as mental events—e.g., “I” raise my hand. While the *suitas* requires the mere psychological presence of the actor during the commission of the crime, the second mode of intentionality—*mens rea* or culpability in Anglo-Saxon systems—refers to the *quality* of that psychological presence, that is, the specific attitude of the perpetrator: the act is culpable only if it is produced by a guilty mind. Rather than being a dichotomous conceptual category, different levels of culpability have been established in all legal systems. For instance, in US criminal law, levels of culpability range from negligent (i.e., the actor does not consider the risk of his or her conduct) to purposeful (i.e., the criminal act is driven by the intention to commit the crime). A third mode of intention is that of the *insanity defense*: in order to be punishable, a crime has to be produced by a healthy and intact mind. It is crucial to note here the subtle distinctive element of the category of the insanity defense as compared to both the *suitas* and the *mens rea.* On the one hand, a crime committed by an insane mind, say during a psychotic episode, cannot be considered an automatism—and therefore there is *suitas*; on the other hand, the criminal act must also be the result of a guilty mind—so there is also *mens rea*. A patient diagnosed with schizophrenia who accidentally shoots the neighbor would be excused by the court because there is no crime; while the same patient who shoots the neighbor during a psychotic episode would be judged not guilty by reason of insanity.

Taken together, these considerations stress that in order to change the law, neuroscientists should first refer to the multifaceted conceptualizations of intentions (e.g., *suitas, mens rea, insanity*) that have been developed within the law throughout the centuries. More advanced theoretical models and new experimental paradigms can be developed on the basis of such categories, so that empirical data can directly feed the juridical discussion about how well these categories describe the nature of human volition. Without this bidirectional contribution, the neuroscientific challenge to free will and personal responsibility will have only limited consequences for the law. In order to appraise the potential revolutionizing impact of neuroscience on the law, it is therefore necessary to understand whether, and how, the law can reframe the neuroscientific study of human volition.

### Conflict of interest statement

The authors declare that the research was conducted in the absence of any commercial or financial relationships that could be construed as a potential conflict of interest.

## Footnotes

^1^Translated by the authors.

^2^One important aspect of the study by Soon et al. (2008) is often ignored, namely that the prediction accuracy is only 60%. This means that in 40% of the trials one cannot predict the choice correctly. This of course leaves a lot of space for free will, and therefore existing data do not exclude the possibility that conscious intentions are sometimes causally effective.

^3^Hence the definition “defense of automatism” in the Anglo-Saxon system.

## References

[B1] Asbury K. Plomin R. (2013). G is for Genes: The Impact of Genetics on Education and Achievement. Chichester: John Wiley & Sons.

[B2] Brass M. Haggard P. (2008). The what, when, whether model of intentional action. Neuroscientist 14, 319–325. 10.1177/1073858408317417 18660462

[B3] Churchland P. S. (2011). Braintrust: What Neuroscience tells Us about Morality. Princeton, NJ: Princeton University Press.

[B4] Frith C. (2007). Making up the Mind: How the Mind Creates Our Mental World. Oxford, UK: Wiley-Blackwell.

[B5] Greene J. Cohen J. (2004). For the law, neuroscience changes nothing and everything. Philos. Trans. R. Soc. Lond. Ser. B 359, 1775–1785. 10.1098/rstb.2004.1546 15590618 PMC1693457

[B6] Haggard P. Eimer M. (1999). On the relation between brain potentials and the awareness of voluntary movements. Exp. Brain Res. 126, 128–133. 10.1007/s002210050722 10333013

[B7] Hallett M. (2007). Volitional control of movement: the physiology of free will. Clin. Neurophysiol. 118, 1179–1192. 10.1016/j.clinph.2007.03.019 17466580 PMC1950571

[B8] Lavazza A. Sammicheli L. (2012). Il Delitto del Cervello. La mente tra scienza e diritto. Torino: Codice Edizioni.

[B9] Libet B. Gleason C. A. Wright E. W. Pearl D. K. (1983). Time of conscious intention to act in relation to onset of cerebral activity (readiness-potential). The unconscious initiation of a freely voluntary act. Brain 106, 623–642. 10.1093/brain/106.3.623 6640273

[B10] Lombroso C. (1876). L'uomo Delinquente in Rapporto All'antropologia, Alla Giurisprudenza ed alle Discipline Carcerarie. Bologna: Il Mulino.

[B11] Meyer-Lindenberg A. Buckholtz J. W. Kolachana B. R. Hariri A. Pezawas L. Weinberger D. R.. (2006). Neural mechanisms of genetic risk for impulsivity and violence in humans. Proc. Natl. Acad. Sci. U.S.A. 103, 6269–6274. 10.1073/pnas.0511311103 16569698 PMC1458867

[B12] Nahmias E. Coates D. J. Kvaran T. (2007). Free will, moral responsibility, and mechanism: experiments on folk intuitions. Midwest Stud. Philos. 31, 214–242. 10.1111/j.1475-4975.2007.00158.x

[B13] Nahmias E. Morris S. Nadelhoffer T. Turner J. (2005). Surveying freedom: Folk intuitions about free will and moral responsibility. Philos. Psychol. 18, 561–584. 10.1080/09515080500264180

[B14] O'Hara E. A. (2004). How neuroscience might advance the law. Philos. Trans. R. Soc. Lond. Ser. B 359, 1677–1684. 10.1098/rstb.2004.1541 15590609 PMC1693456

[B15] Rigoni D. Brass M. Roger C. Vidal F. Sartori G. (2013). Top-down modulation of brain activity underlying intentional action and its relationship with awareness of intention: An ERP/Laplacian analysis. Exp. Brain Res. 229, 347–357. 10.1007/s00221-013-3400-0 23354661

[B16] Sellars W. (1963). Philosophy and the scientific image of man. Sci. Percept. Real. 2, 35–78.

[B17] Shariff A. F. Greene J. D. Karremans J. C. Luguri J. B. Clark C. J. Schooler J. W.. (2014). Free will and punishment: a mechanistic view of human nature reduces retribution. Psychol. Sci. 1, 1–8. 10.1177/0956797614534693 24916083

[B18] Soon C. S. Brass M. Heinze H. J. Haynes J. D. (2008). Unconscious determinants of free decisions in the human brain. Nat. Neurosci. 11, 543–545. 10.1038/nn.2112 18408715

[B19] Wegner D. M. (2002). The Illusion of Conscious Will. Cambridge: MITpress.

